# Editorial: Personality and Cognition in Economic Decision Making

**DOI:** 10.3389/fpsyg.2017.00848

**Published:** 2017-05-23

**Authors:** Aurora García-Gallego, Manuel I. Ibáñez, Nikolaos Georgantzis

**Affiliations:** ^1^Laboratorio de Economía Experimental and Economics Department, Universitat Jaume ICastellón, Spain; ^2^Department of Basic and Clinical Psychology, Universitat Jaume ICastellón, Spain; ^3^School of Agriculture Policy and Development, University of ReadingReading, United Kingdom

**Keywords:** personality, cognition, decision making

Recently, psychologists studying cognitive processes and personality have increasingly benefitted from the wealth of theory, methodology, and decision making paradigms used in economics and game theory. Similarly, for the economists, personality traits and basic cognitive processes offer a set of coherent explanatory constructs in economic behavior. Given the debate on preference invariance and behavioral consistency across contexts and domains, the papers in this topic shed light on the existence and effect of stable sets of idiosyncratic features on economic decision-making.

In Waskow et al., PWYW decisions are studied while acquiring FMRI data. Participants buy music either under a traditional “fixed-price” (FP) condition or under a PWYW mechanism. The data replicate previous results on the general feasibility of the PWYW mechanism. In the FP-condition, neural activity in frontal areas during decision-making correlates positively with the participants' willingness to pay. No such relationship was observed under PWYW in any neural structure. Stronger activity of the lingual gyrus was observed during PWYW.

In Proestakis and Brañas-Garza, the authors deal with the degree to which obese people adjust their own behavior as a result of anticipated discrimination. Consistent with the System Justification Theory, the study finds that self-identified obese individuals request lower amounts of money. Self-perceived but not externally reported excessive weight captures the self-weight bias not only for obese but also for non-obese individuals. This self-weight bias, yielding lower salary requests, enhances discriminatory behavior against individuals who feel, but may not actually be, obese and consequently exacerbates the wage gap.

Corgnet et al. studies whether the push for recruiting diligent millennials using criteria such as cognitive reflection can ultimately hamper the recruitment of creative workers. A positive effect is observed of fluid intelligence on originality and elaboration measures of divergent creative thinking. Furthermore, the U-shape relationship between cognitive reflection and fluency and flexibility measures of divergent creative thinking is inverted. This suggests that thinking too much may hinder important dimensions of creative thinking. Diligent and creative workers may thus be rare.

In Zhu et al., event-related potentials were recorded to evaluate brain responses when gambling for individual self, a close friend (relational self), or a class (collective self). When outcome feedback was positive, gambling for the individual “self” evoked a larger reward positivity compared with gambling for a friend or for the class, while there is no difference between the latter two conditions. When outcome feedback was negative, no significant effect was found between conditions. These findings provide direct electrophysiological evidence that the individual self is at the top of the three-tier hierarchy of the motivational system in the collectivist brain.

In Shang et al., it is observed that the choice effect is a robust phenomenon in which even “mere choice,” not including actual actions could intensify the preference for the self-chosen over other-chosen objects. Two studies examine the hypothesis. The results showed that the mere choice effect measured by Implicit Association Test (IAT) significantly decreased for participants with lower levels of trait autonomy (Study 1) and when participants were primed to experience autonomy deprivation (Study 2).

In Radell et al., a novel computer-based CPP task is developed in which participants guide an avatar to enter into a room with frequent (i.e., rich) and less frequent (i.e., poor) rewards. Low IU individuals enter into both rooms at about the same rate, while high IU individuals enter into the previously rich room first. The latter's attraction to rewards is consistent with previously observed behavior in opioid-addicted individuals. Thus, high IU may lead to a cognitive bias favoring increased vulnerability to addiction.

In Itzkin et al., the participants received six decision scenarios, in which they were asked to evaluate regret following action and inaction. Individual regulatory focus was measured by two scales. Promotion-focused individuals attributed less regret than prevention-focused individuals to action decisions. Regret following inaction was not affected by regulatory focus. In addition, a trigger for change decreases regret following action. Orthodox people tend to attribute more regret to an action decision. Thus, both the situation and a decision maker's orientation affects regret after action and inaction.

In Ring et al., the performance predictions in the 7-item Cognitive Reflection Test (CRT) is studied. After completing the test, subjects predicted their own, other participants', men's, and women's, correct answers. Men scored higher on the CRT than women and both men and women were too optimistic about their own performance. However, men think they perform significantly better than other men and do so significantly more than women. The equality between women's predictions about their own performance and their female peers cannot be rejected.

In Alós-Ferrer et al., novel evidence is presented on response times and personality traits in standard questions from the decision-making literature where responses are relatively slow (medians around half a minute or above). All questions create a conflict between an intuitive process and more deliberative thinking. For CRT questions, the differences in response times are as predicted by dual-process theories, with alignment and heuristic variants leading to faster responses and neutral questions to slower responses than the original, conflict questions. For decision biases (where responses are slower), evidence is mixed.

In Hanaki et al., the authors study the relationships between the key facets of dominance solvability and two cognitive skills, cognitive reflection, and fluid intelligence. Dominance and one-step iterated dominance are both predicted by one's fluid intelligence rather than cognitive reflection. Individual cognitive skills, however, only explain a small fraction of the observed failure of dominance solvability. The accuracy of theoretical predictions on strategic decision making thus not only depends on individual cognitive characteristics, but also, perhaps more importantly, on the decision making environment itself.

Terzi et al. investigates the capacity of four potential reference points—(1) population average payoff, (2) announced expected payoff of peers in similar situations, (3) a historical average of earnings in the same task, and (4) an announced anticipated individual payoff—to organize decisions in a risky decision making task. The population average payoff is the modal reference point, followed by experimenter's stated expectation of individual earnings, followed by average earnings of other participants. A sizeable share of individuals show multiple reference points. The reference point is not affected by a shock to her income.

In Myrseth and Wollbrant, the association between “intuitive” and “fast” (Cappelen et al., 2015) is discussed. The commentary argues that such an association requires “fast” to rule out “deliberative,” which would need information beyond relative response speed. The precise cut-off time for deliberative decisions may be difficult to establish (see e.g., Schneider and Shiffrin, 1977; Posner and Rothbart, 1998), thus, an individual offered a few seconds, may still have sufficient time to reflect consciously. Thus, “faster” responses ought not to be taken as “intuitive” prima facie.

In Breaban et al., an experiment is run to consider the emotional correlates of prudent decision making. Subjects were presented with lotteries, while their emotional responses were recorded with facial recognition software. They had to make binary choices between risky lotteries that distinguish prudent from imprudent individuals. They also perform tasks designed to assess their cognitive ability and a number of personality characteristics. It is found that a more negative emotional states correlate with greater prudence. Higher cognitive ability and less conscientiousness are also associated with greater prudence.

In Bejarano et al., independently reported measures of subjects' cognitive capabilities, preferences, and sociodemographic characteristics relate to behavior in a real-effort moral dilemma. Rather than simple correlation, clustering subjects into groups based on behavior in the real-effort task reveals important systematic differences across groups. However, the results indicate a need for a more comprehensive theory explaining how combinations of different individual characteristics impact behavior.

In Barreda-Tarrazona et al., four different groups of subjects are created based on subjects' scores in altruism and reasoning ability. Subjects play both one-shot (random changing pairs) and repeated (fixed partners) prisoner's dilemma (PD) games. Incentivised beliefs regarding cooperation are elicited, showing that high altruism leads to optimism about others' cooperation and higher cooperation in the first repetitions of PD. Contrary to the one-shot PD, high reasoning ability increases the probability of cooperation.

In Wei et al., individual differences are combined with social influence, revealing the effect of social value orientation (SVO) and social influence on prosocial behavior in trust and dictator experiments. In the trust game, prosocials were less likely than proselfs to conform to other members' behavior, when the majority of group members distrusted the trustee. In the dictator game, prosocial subjects were influenced more by others' generous choices than their selfish choices, even if the latter benefitted them. The results indicate that the effect of social influence appears to depend on individuals' SVO.

In Zhao et al., two studies examine individual differences in two forms of prosociality—generosity and reciprocity—with respect to two major models of personality, the Big Five and the HEXACO. Both generosity and positive reciprocity determine social preferences. Men were more generous when this was costless and women were more egalitarian overall. HEXACO honesty–humility predicted dictator, but not generosity allocations, while irritability and anger predicted lower generosity, but not dictator allocations. Politeness of Big Five agreeableness was uniquely and broadly associated with prosociality across all games.

Zhao et al. examines the association between the Dark Triad of personality (i.e., Machiavellianism, narcissism, and psychopathy) and corruption. The positive relation between the Dark Triad and bribe-offering or bribe-taking intention was mediated by the belief in good luck. Therefore, belief in good luck may be one of the reasons explaining why people with Dark Triad are more likely to engage in corruption regardless of the potential outcomes.

Ibáñez et al. studies the association among different sources of individual differences such as personality, cognitive ability, and risk attitudes with trust and reciprocity in an incentivized binary trust game. Trust associates to positive urgency and emotionality and, specifically, to the extraversion's warmth facet. Participants scoring high in psychopathy exhibit increased electrodermal activity and reduced evoked heart rate deceleration when asked to decide whether or not to trust. Abstract reasoning and low disagreeable disinhibition favor reciprocity, while lack of reciprocity relates with a psychopathic, highly disinhibited, and impulsive personality.

While the effects of personality and cognition on economic decisions remain underexplored, the papers contributed in this topic offer more than a stimulus for further research. The general message could be that personality and cognitive processes offer the stable idiosyncratic ground on which individual decisions are made.

## Author contributions

All authors listed, have made substantial, direct and intellectual contribution to the work, and approved it for publication.

### Conflict of interest statement

The authors declare that the research was conducted in the absence of any commercial or financial relationships that could be construed as a potential conflict of interest.
